# Dietary Oleic Acid Increases M2 Macrophages in the Mesenteric Adipose Tissue

**DOI:** 10.1371/journal.pone.0075147

**Published:** 2013-09-30

**Authors:** Christina Camell, C. Wayne Smith

**Affiliations:** 1 Department of Pathology and Immunology, Baylor College of Medicine, Houston, Texas, United States of America; 2 Department of Pediatrics, Baylor College of Medicine, Houston, Texas, United States of America; 3 Children’s Nutrition Research Center, Houston, Texas, United States of America; Virginia Tech, United States of America

## Abstract

Several studies have implicated fatty-acids as inflammatory regulators, suggesting that there may be a direct role for common dietary fatty-acids in regulating innate immune cells. In humans, a single high-fat meal increases systemic cytokines and leukocytes. In mice, short term high-fat feeding increases adipose tissue (AT) leukocytes and alters the inflammatory profile of AT macrophages. We have seen that short term high fat feeding to C57BL/6J male mice increases palmitic and oleic acid within AT depots, but oleic acid increase is highest in the mesenteric AT (MAT). In vitro, oleic acid increases M2 macrophage markers (CD206, MGL1, and ARG1) in a murine macrophage cell line, while addition of palmitic acid is able to inhibit that increase. Three day supplementation of a chow diet, with oleic acid, induced an increase in M2 macrophage markers in the MAT, but not in the epididymal AT. We tested whether increases in M2 macrophages occur during short term *ad lib* feeding of a high fat diet, containing oleic acid. Experiments revealed two distinct populations of macrophages were altered by a three day high milk-fat diet. One population, phenotypically intermediate for F4/80, showed diet-induced increases in CD206, an anti-inflammatory marker characteristic of M2 macrophages intrinsic to the AT. Evidence for a second population, phenotypically F4/80^HI^CD11b^HI^ macrophages, showed increased association with the MAT following short term feeding that is dependent on the adhesion molecule, ICAM-1. Collectively, we have shown that short term feeding of a high-fat diet changes two population of macrophages, and that dietary oleic acid is responsible for increases in M2 macrophage polarization.

## Introduction

Adipose tissues (AT) contain resident populations of macrophages and the phenotypic characteristics of these cells can be influenced by dietary factors. For example, in murine models of diet-induced obesity, epididymal adipose tissue (EAT) macrophages prominently express pro-inflammatory genes [1]. These changes are evident after weeks of feeding high fat diets. In contrast, EAT in lean animals contain macrophages primarily characterized as anti-inflammatory [1], [2]. Saturated fat has been implicated in the induction of pro-inflammatory changes in adipose tissue macrophages during long term feeding studies [3]. Furthermore, studies in vitro have shown direct stimulation of pro-inflammatory cytokine gene expression in adipocytes and macrophages by palmitic acid, apparently signaling through toll-like receptor (TLR)-4 [4]. However, the complex cascades of the evolving inflammatory response in vivo to obesogenic diets confound understanding of simple cause and effect relationships.

Some investigations have relied on short-term feeding of high fat diets in an effort to assess early, possibly initiating events in diet-induced adipose tissue inflammation. In humans, a single high fat meal transiently changes plasma cytokine and lipid profile, increases peripheral blood leukocytes, and impairs vasodilation [5]. In mice, short term feeding of a 60% lard diet has been reported to increase AT macrophages [6] and neutrophils [7], and to alter the inflammatory profile of AT macrophages [8]. It is of interest that the phenotypic changes in macrophages in the short term feeding studies do not mirror those in the long term feeding studies. Given a finding that oleic acid, another prominent component of the high fat diets, inhibits a wide variety of inflammatory inducers in vitro [9], [10], and binds PPARγ [11], a transcription factor which promotes polarization of anti-inflammatory AT macrophages [12], [13], we chose to focus on possible effects of this fatty acid on macrophages in murine AT.

In this report, we concentrate on macrophages in the peritoneal cavity and in two distinct collections of adipose tissue in the abdominal cavity, the EAT and mesenteric AT (MAT). The MAT surrounds the intestines, contains lymphatics for triglyceride entry into the blood and is more closely related to the human intra-abdominal depot (omentum). We show that dietary fatty-acids induce changes in both MAT and peritoneal macrophages, that oleic acid ingestion induces M2 polarization of the AT macrophages.

## Methods

### Ethics Statement

This study was carried out in strict accordance with the recommendations in the Guide for the Care and Use of Laboratory Animals of the National Institutes of Health. The protocol was approved by the Institutional Animal Care and Use Committee of Baylor College of Medicine (Animal Welfare Assurance number: 3823-01).

### Animals and Tissue Preparation

Male C57BL6/J mice were purchased at 5 wks of age (Jackson Laboratory), allowed to adapt, and fed at 6–7 wks of age. *Icam-1−/−,* and *Mcp-1−/−* were bred in our facility and fed at 6–7 wks of age. Control (Harland Teklad, #2020; kcal%: fat 16%, carbohydrate 62%, protein 22%), 42% fat (Dyets, etc. #102457; kcal %: fat 42%, carbohydrate 42%, protein 16%) and 60% fat (Dyets, #102784; kcal %: fat 60%, carbohydrate 26%, protein 14%) were purchased for feeding studies. To asses response to 42% milk-fat diet, indirect calorimetry, food consumption and activity levels were assessed at the Children’s Nutrition Research Center Mouse Metabolic Research Unit using the comprehensive laboratory animal monitoring system (Columbus Instruments) for a cohort of mice (n = 5) after acclimatization to single housing and a new cage environment. For tissue collection and analysis after 3 days of control or milk-fat diet, mice were anesthetized, and whole-body perfusion was performed by cardiac puncture. Tissue was harvested for freezing in liquid nitrogen and storage at −80°C (real time PCR analysis) or storage in 2% BSA in PBS (flow cytometry analysis). Peritoneal cells were collected by lavage using 5 ml of ice cold PBS. In some experiments adipose tissue was washed with PBS to remove adherent cells.

### Real Time PCR

Adipose tissue was homogenized using a mini bead beater (Biospec Products, Bartlesville, OH). Total RNA was isolated using Rneasy columns (Qiagen #74804) according to manufacturer’s instructions. 500 ng of RNA was reversed transcribed into cDNA (AMV reverse transcriptase; Roche) for qPCR using Taqman probes. Gene expression was normalized to the housekeeping gene GAPDH.

### ELISA

Protein was isolated from adipose tissue stored at −80°C by homogenizing tissue in RIPA buffer containing protease inhibitors. MCP-1 levels were measured according to manufacturer’s instructions (R&D systems). Total protein levels were determined using a Bradford assay (Bio-Rad Laboratories, Inc).

### Stroma Vascular Fraction Isolation and Flow Cytometry

As previously described [14], adipose tissue from 3 mice was pooled for each preparation. MAT or EAT was excised, weighed, minced into small pieces and placed into PBS containing 2%BSA. Samples were digested in collagenase I (Worthington Chemicals), at 280 U/mL for 45 min at 37°C. They were filtered in chiffon to remove large particles and centrifuged to separate adipocytes from the stroma vascular fraction (SVF). SVF was washed in PBS and stained using desired antibodies. Antibodies used include: F4/80 (APC; BM8; eBioscience; 17-4801-82), CD206 (FITC; MR5D3, AbD Serotec; MCA2235F), Rat IgG2a (APC; BD Pharmingen; 553932), CD11b (PE; Millipore; CBL1313P), Rat IgG2a (FITC; AbD Serotec), Rat IgG2a (PE; BD Pharmingen). Cells were fixed in BD FACS Lysing Solution (BD Biosciences) and CountBright counting beads (Invitrogen) were added immediately prior to analysis on an LSRII Flow Cytometer (BD Biosciences).

### Fatty-acid Culture

RAW 264.7 (American Tissue Cell Company, ATCC) cells were maintained in DMEM (Sigma; D6429) containing 10% FBS. Cells were plated at 250,000 cells/well in a 24 well-plate and serum starved overnight in low-glucose DMEM. Cells were treated for 20 additional hours in low glucose DMEM containing 1% fatty-acid free BSA (Sigma) and 0.02% ethanol or purified fatty-acid (Sigma) dissolved in ethanol and complexed to fatty-acid free BSA. Cells treated with media alone were used as a control to test for endotoxin contamination. Media and BSA treated cells had similar levels of TNFα expression indicating there was no endotoxin contamination through the BSA preparation. Total RNA was isolated (RNeasy; Qiagen) and gene expression analyzed by Taqman qPCR. Fold change was calculated using BSA as a baseline using the comparative CT method.

### Bone Marrow Derived Macrophages

BMDMs were generated as described before [15]. In brief, bone marrow was harvested from wild-type mice and cultured for 7 days in RPMI 1640 containing 10% FBS, 10% Antibiotic Antimycotic Solution (Sigma-Aldrich) and 20% L929 cell-conditioned media at 37°C in 5%CO_2_ atmosphere. Additional 5 ml of culture medium was added every second day. On the seventh day, BMDMs were washed with PBS and plated at 250,000 cells/well for fatty-acid treatment similar to that described above, with one change: control and fatty acid treatments were prepared in RPMI 1640 and not DMEM.

### Lipid Analysis

AT samples were harvested, frozen in liquid nitrogen and stored at −80°C prior to analysis by gas chromatography at MMPC/DRTC Lipid Lab in Vanderbilt (DK59637).

*Oral Gavage Feeding:* This protocol was slightly modified from one previously reported [16]. At 6–7 weeks of age C57BL6/J male mice were given a twice daily oral gavage containing 0.3 g diet powder (Dyets, etc. #710125) and 400 ul of water or 0.3 g diet powder, 0.3 g fatty-acid and 300 ul of water. The amount of fatty-acid given is similar to the amount of total fatty-acid eaten by mice on 42% HMF diet, and was determined using data from food intake experiments. Mice had access to chow (Harland Teklad, #2020) and water *ad lib*. After three days, mice were sacrificed and adipose harvested for analysis.

### Data Analysis

Values are presented as means ±SD. Results were analyzed using a 2-tailed Student’s *t* test, or 1-way ANOVA as appropriate, using GraphPad Prism Software. Significance was accepted at P<0.05.

## Results

### High Milk Fat (HMF) Diet Increases Oleic Acid in Abdominal AT

**Figure 1:**
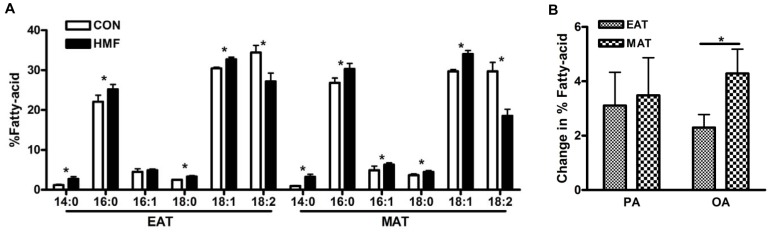
Oleic acid increase is highest in MAT. Mass spectrometry analysis of (A) fatty-acid composition (expressed as a percentage of total fatty acid) in the EAT and MAT following three control or 60% HMF feeding. (B) Change in the percentage of palmitic (PA) and oleic (OA) acid out of total fatty-acids expressed as the difference between control and HMF-fed mice. (N = 5–6).

To test which fatty-acids are most prominent within the AT following HMF feeding, mice were fed control or HMF diet for three days. Here, we focus on palmitic (16∶0) and oleic (18∶1) acids, as those are most common within milk-fat (Table 1). Palmitic and oleic acid make up a large percentage of AT fatty-acids on control feeding and this amount is increased following HMF feeding (Figure 1A). The percent increase in oleic acid is greater in mesenteric adipose tissue (MAT) following HMF feeding, than in epidydimal adipose tissue (EAT), while the percent increase in palmitic acid was the same in both AT depots (Figure 1B). Oleic acid makes up the largest percentage of MAT fatty-acids following HMF feeding (Table 2).

**Table 1:** Fatty acid composition of milk-fat.

Fatty-Acid		% of total FA
Butric	4∶0	3.9
aproic	6∶0	2.4
Caprylic	8∶0	1.2
Capric	10∶0	2.8
Lauric	12∶0	3.2
Myristic	14∶0	10.4
Palmitic	16∶0	28.2
Stearic	18∶0	12.2
Oleic	18∶1	21.5
Linoleic	18∶2	2.8
Other		11.8
Saturated		67.7
Monounsaturated		24.7
Polyunsaturated		3.7
Omega-3		0.6
Omega-6		3.2

Modified from Dyets Inc.

**Table 2:** Fatty acid composition in adipose tissues following short term control or HMF feeding.

		Fatty-acid total (%)			Chg in % of FA			
	CON EAT	HMF EAT	CON MAT	HMF MAT	EAT	MAT	P	MAT:EAT
14∶0	1.2±0.2	2.7±0.5	0.9±0.1	3.3±0.6	1.5±0.5	2.3±0.6	0.03	**↑**
16∶0	22.1±1.6	25.2±1.2	26.8±1.2	30.3±1.4	3.1±1.2	3.5±1.4	0.63	**NS**
16∶1	4.5±0.8	4.9±0.3	4.9±1.0	6.3±0.5	0.4±0.3	1.3±0.5	0.00	**↑**
18∶0	2.5±0.1	3.3±0.2	3.7±.33	4.5±0.3	0.8±0.2	0.8±0.3	0.97	**NS**
18∶1w9	30.5±0.3	32.8±0.5	29.7±0.45	34.0±0.9	2.3±0.5	4.3±0.9	0.00	**↑**
18∶1w7	1.5±0.2	1.2±0.1	1.7±0.15	1.4±0.2	−0.3±0.1	−0.4±0.2	0.64	**NS**
18∶2	34.4±1.8	27.2±2.1	29.7±2.2	18.55±1.6	−7.2±2.1	−11.2±1.6	0.00	**↓**
18∶3 w3	2.3±0.1	1.8±0.2	1.8±0.1	1.1±0.1	−0.5±0.2	−0.7±0.1	0.08	**NS**
20∶3w6	0.2±0.03	0.2±0.04	0.2±0.04	0.2±0.01	−0.02±0.04	−0.05±0.01	0.11	**NS**
20∶4	0.4±0.05	0.4±0.05	0.4±0.06	0.3±0.02	−0.08±0.05	−0.12±0.02	0.05	**↓**
20∶5	0.02±0.04	0.04±0.03	0.00	0.00	0.01±0.03	0.00	0.32	**NS**
22∶5w3	0.1±0.01	0.09	0.00	0.02±0.03	−0.01±0.02	0.02±0.04	0.12	**NS**
22∶6	0.2±0.03	0.17±0.04	0.068±0.09	0.1±0.02	−0.04±0.04	0.06±0.02	0.00	**↑**

(TOP) Percentage of individual fatty-acids within adipose depots following control (CON) or high milk fat (HMF) feeding. (BOTTOM) Change in percentage of fatty-acids (FA) is calculated by subtracting control values from HMF values. P value represents a T test comparing changes in percentage of fatty-acids between depots. NS = non-significant. **↑** = significantly increased in MAT. **↓** = significantly decreased in MAT.

### Oleic Acid Increases M2 Macrophage Markers in vitro

**Figure 2:**
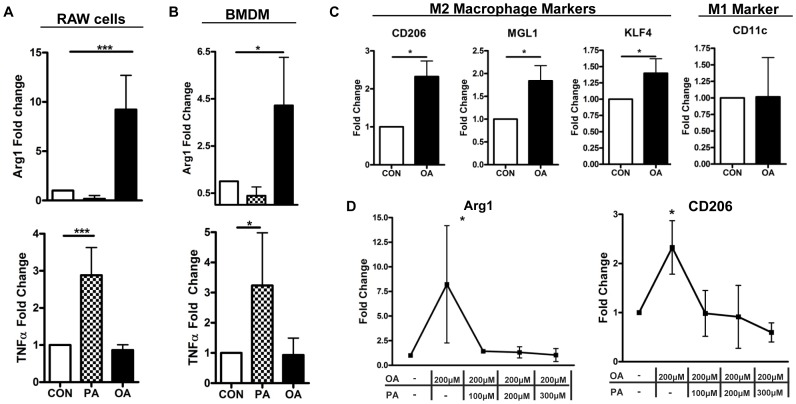
Oleic acid induces M2 macrophage markers in Arginase-1 (Arg1) and TNFα gene expression fold change from (A) RAW 264.7 macrophages or (B) bone marrow derived macrophages (BMDM) cultured overnight with oleic (OA) or palmitic (PA) acid (500 µM). (N = 3,4) (C) CD206, MGL1 and KLF4 gene expression fold change in RAW 264.7 macrophages following overnight culture in OA (500 µM). (D) Arg1 and CD206 gene expression fold change from RAW 264.7 macrophages cultured in OA (200 µM) and PA (as labeled). Control cells were cultured in BSA and ethanol. (N = 3).

In-vitro, macrophages have increased Arginase1 (Arg1) expression following treatment with a fatty-acid mixture that is mainly composed of oleic acid [17]. To test if oleic acid alone can upregulate Arg1 gene expression in macrophages, a murine macrophage cell line (RAW 264.7) was cultured overnight in oleic acid complexed to fatty-acid free BSA. Arg1 expression was significantly upregulated by oleic acid treatment, whereas palmitic acid did not effect Arg1 expression (Figure 2A). As previously seen in the literature [18], palmitic acid upregulated TNFα expression, while oleic acid treatment did not (Figure 2A). These results were confirmed in bone marrow derived macrophages (Figure 2B). M2 macrophage markers: MGL1, CD206 and KLF4, were all significantly increased in macrophages treated with oleic acid as compared to control treated only with BSA (Figure 2C). There was no change in CD11c, a classical M1 macrophage marker (Figure 2C). To test for the effects of palmitic acid on oleic acid, increasing amounts of palmitic acid were added with 200 µM of oleic acid for overnight culture. At 100 µM, the lowest concentration of palmitic acid added, there was complete inhibition of oleic acid-induced Arg1 and CD206 (Figure 2D).

### Oleic Acid Increases M2 Macrophage Markers in Mesenteric Adipose Tissue

**Figure 3:**
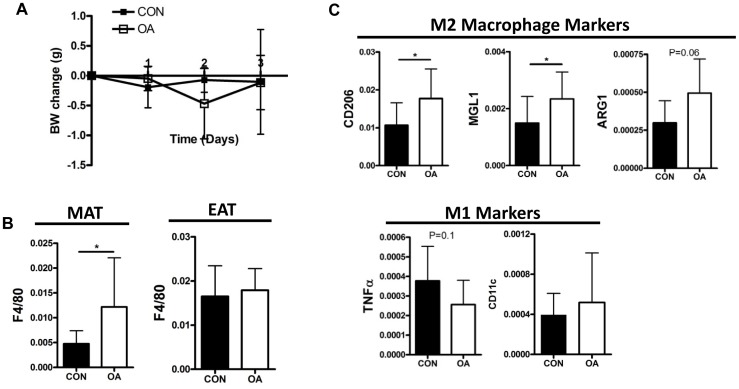
Dietary oleic acid increases M2 macrophage markers in the MAT. (A) Change in body-weight during a three day, twice daily, oral gavage with control or purified OA. (B) F4/80 gene expression (qPCR relative to GAPDH) in mesenteric adipose tissue (MAT) or epididymal adipose tissue (EAT) from mice given control or OA gavage. (C) Gene expression (qPCR relative to GAPDH) analysis of M2 and M1 macrophage markers in MAT from gavaged mice. (N = 10, 12).

To test whether dietary fatty-acids can affect ATMs, control chow was supplemented with purified oleic or palmitic acid and given by oral gavage, twice daily, for three days. There were no differences in weight in the control, PA-gavage or OA-gavage mice (Data not shown and Figure 3A). Expression of F4/80, a macrophage marker, was increased in MAT, but not EAT (Figure 3B). Consistent with in vitro observations, M2 macrophage markers (CD206 and MGL1), but not M1 macrophage markers, TNFα or CD11c, were increased following OA-gavage in the MAT (Figure 3D). No changes in M1 or M2 macrophage markers were identified in the MAT following PA gavage (data not shown).

### Metabolic and Body Composition Changes in Response to 3 Day Milk-fat Diet

**Figure 4:**
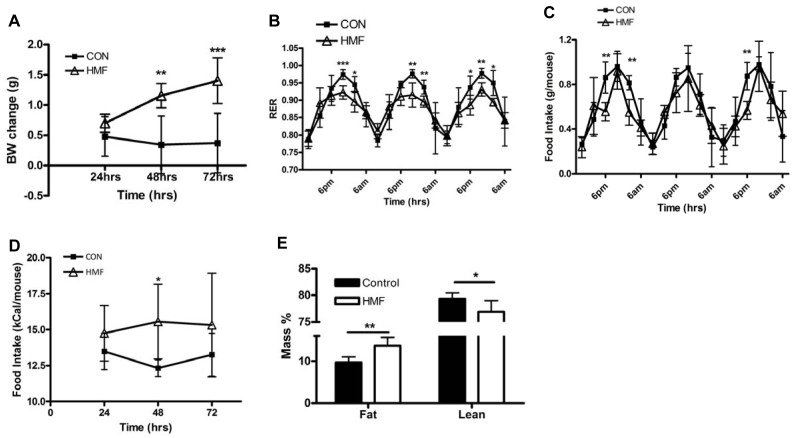
Metabolic and body composition changes in response to 3 day HMF feeding. The mouse metabolic research unit was utilized to analyze (A) body-weight changes, (B) respiratory exchange ratio (RER) and (C&D) food-intake in mice fed a three day control or 42% HMF diet. (N = 5) (E) Fat and lean mass were measured using MRI. (N = 5).

Mice were fed a three day control or HMF diet to test whether a diet containing mixed fats, rich in oleic acid (table 1) could induce M2 macrophage marker gene expression changes in adipose tissue. Body-weight, food intake and metabolic activity were analyzed to ensure mice were eating the diet. The 3 day, HMF diet significantly increased body-weight compared to control feeding (Figure 4A). Respiratory exchange ratio (RER) significantly decreased in HMF feeding between 10 pm and 2 am, the murine active time. This is consistent with metabolizing the higher fat diet. During the light hours, (6 am–6 pm), there were no significant differences between the control fed and HMF fed mice (Figure 4B). The volume of food intake was similar between mice fed a control or HMF diet, although at certain time points control fed mice ate more than HMF-fed (Day 1∶6 pm and 2 am and Day 3∶6 pm) (Figure 4C). The kCal/mouse intake was significantly higher in the HMF-fed mice during the second day of feeding, although no differences were seen on day one or three (Figure4D). No differences in activity levels were found over the three-day period (data not shown). Control and HMF-fed mice were analyzed by qMRI for body-composition changes. HMF feeding significantly increased fat mass percentage and decreased lean mass percentage (Figure 4E).

### The HMF Diet Alters Macrophage Populations in the Mesenteric Adipose Tissue

**Figure 5:**
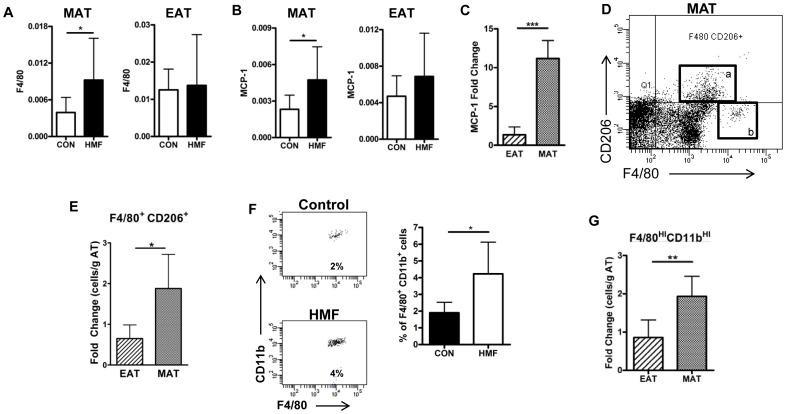
Diet-induced macrophage changes in MAT and EAT. (A) F4/80 expression (qPCR relative to GAPDH) in MAT and EAT (n = 4–8). (B) MCP-1 expression (qPCR relative to GAPDH) in MAT and EAT (n = 4–8). (C) Changes in MCP-1 protein detected by ELISA in extracts of adipose tissues and expressed as ratios of HMF diet over control diet adipose tissue levels in EAT and MAT. (n = 4–8) (D) Typical flow cytometry pattern of the SVF of the MAT showing (a) F4/80^Int^ CD206^+^ and (b) F4/80^HI^ CD206^−^ macrophage populations. (E) Changes in the F4/80^Int^ CD206^+^ macrophage population expressed as ratios of HMF diet over control diet adipose tissue cells/gm in EAT and MAT (n = 6). (F) Representative flow cytometry of the F4/80^HI^ CD206^−^ macrophage population in MAT showing CD11b expression with control and HMF diets; and graph of the percentage of these cells in the total macrophage population within the stromavascular fraction of MAT from control and HMF diets. (G) Changes in the F4/80^HI^ CD206^−^ CD11b^Hi^ macrophages expressed as ratios of cells/g in HMF diet over control diet EAT and MAT.

After 3 days on the HMF diet, F4/80 expression was significantly increased in MAT but not EAT (Figure 5A). A prominent chemokine known to attract macrophages was also increased in the MAT but not the EAT (Figure 5B and C). Flow cytometry of the stromavascular fraction of these adipose tissues revealed at least 3 populations of macrophages, F4/80^int^CD206^+^ (Figure 5Da) F4/80^Hi^CD206^−^ (Figure 5Db), F4/80^int^CD206^−^. The F4/80^int^CD206^+^ population significantly increased in the MAT of mice on the HMF diet (Figure 5E), as compared to the EAT. The F4/80^Hi^CD206^−^ population was also found to express high levels of CD11b, another macrophage marker in mice (Figure 5F), and was significantly increased in the MAT of mice on the HMF diet (Figure 5G), but did not change in the EAT. The F4/80^int^CD206^−^ population did not significantly change in either the MAT or EAT. Thus, it appears that the HMF diet increases two populations of macrophages in the MAT, M2 (CD206^+^) macrophages and a distinct population of F4/80^Hi^CD206^−^ CD11b^Hi^ macrophages.

### HMF Diet Induces Peritoneal Cell Adhesion

**Figure 6:**
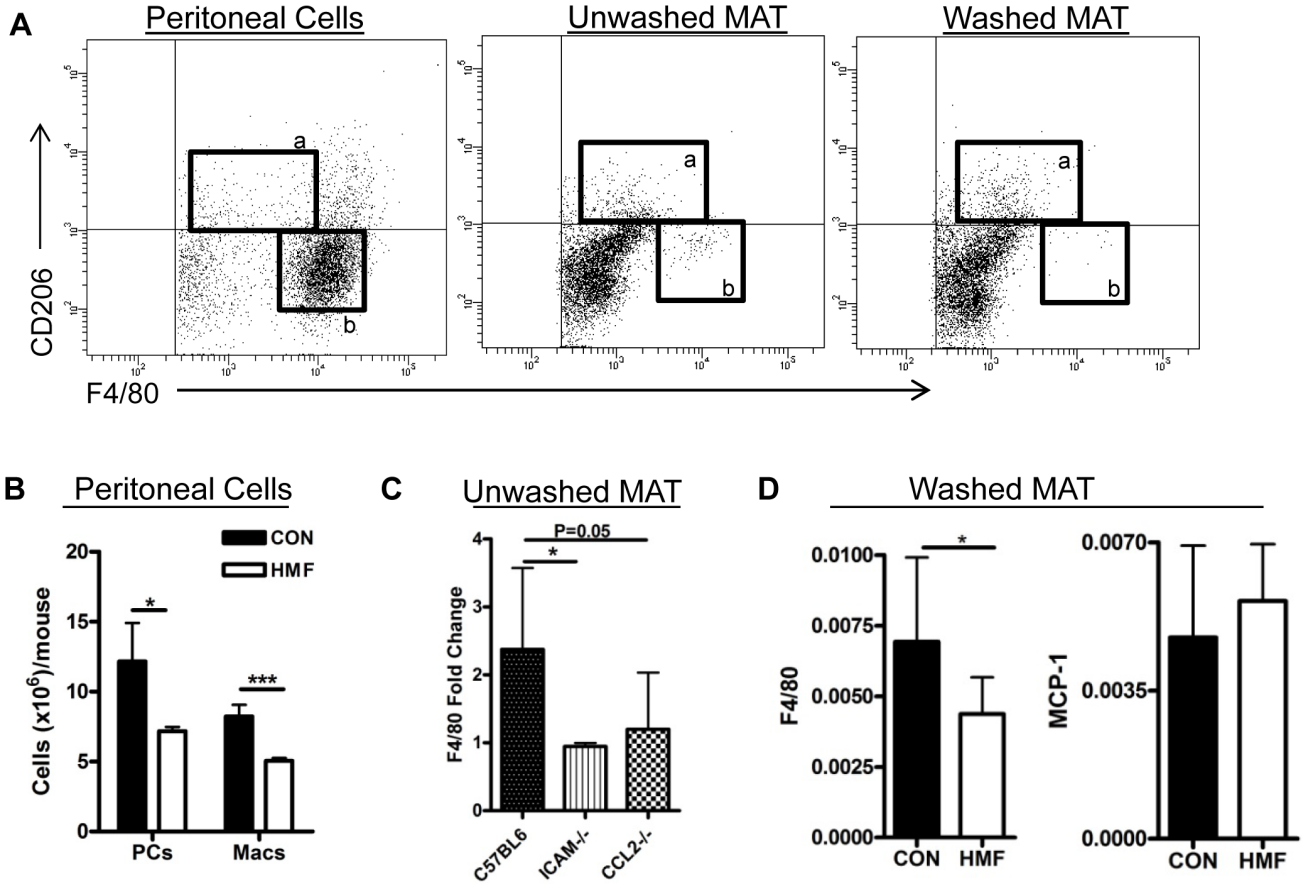
HMF diet induces peritoneal cell adhesion. (A) F4/80 and CD206 expression, by flow cytometry, on peritoneal macrophages, and SVF macrophages isolated from unwashed or washed MAT. F4/80^Int^ CD206^+^ (a) and F4/80^HI^ CD206^−^ (b) macrophage populations are boxed. (B) Total cell counts (per mouse) of peritoneal cells (PCs) or macrophages (Macs) isolated by peritoneal lavage following three day control or milk-fat feeding. (N = 3) (C) C57BL6, MCP-1−/−, or ICAM-1−/− mice were fed control or HMF diet. F4/80 gene expression from HMF unwashed MAT is expressed as fold change relative to mice on control diet. (N = 5–8) (D) F4/80 and MCP-1 gene expression (qPCR, relative to Gapdh) in the washed MAT of mice fed a three day control or HMF diet. (N = 10,11).

Mesenteric adipose tissue is contained within the abdominal cavity and is surrounded by the peritoneal cavity, which contains leukocytes, primarily B cells and macrophages [19]. Peritoneal macrophages expressed high levels of F4/80 (Figure 6Ab) and CD11b (data not shown), but have little expression of CD206. When the MAT that was collected was thoroughly washed to remove any adherent and non-tissue cells, the F4/80^HI^CD206^−^ population of cells was removed (Figure 6Ab), but F4/80^INT^CD206^+^ macrophages were not (Figure 6Aa). Furthermore, animals on the HMF diet had a 50% decrease in total peritoneal cells (PCs) and total F4/80^+^SiglecF^−^ macrophages (Macs) as compared to control fed mice (Figure 6B).

To pursue the possibility that HMF diet induces an increase in peritoneal cell adhesion to MAT, we tested whether an adhesive molecule and a chemotactic factor are required for diet-induced F4/80 increases. Intercellular adhesion molecule (ICAM)-1, a ligand for CD11b, is expressed in the adipose tissue and on mesothelial cells, and is upregulated after a 3-week high-fat diet [20]. When ICAM-1 deficient mice (*Icam-1^−/−^*) were placed on the three day HMF diet, the expected increase in F4/80 expression in the MAT failed to occur as compared to wild-type C57BL6J on the HMF diet (Figure 6C). MCP-1-deficient mice (*Ccl2^−/−^*) had reduced F4/80 expression, although not significant as compared to C57BL6/J on HMF diet (Figure 6C). These results indicate ICAM-1 is required for F4/80 expression increases in the unwashed MAT.

To test whether removal of adherent cells could remove the diet-induced increases in F4/80 and MCP-1 gene expression seen in the unwashed MAT (Figure 5A&B), gene expression in washed MAT was examined following three days control or HMF diet. There was no diet-induce increase (Figure 6D), suggesting that adherent peritoneal cells are the source of HMF-induced changes.

### Dietary Oleic Acid Increases M2 Macrophage Markers in the Washed MAT

**Figure 7:**
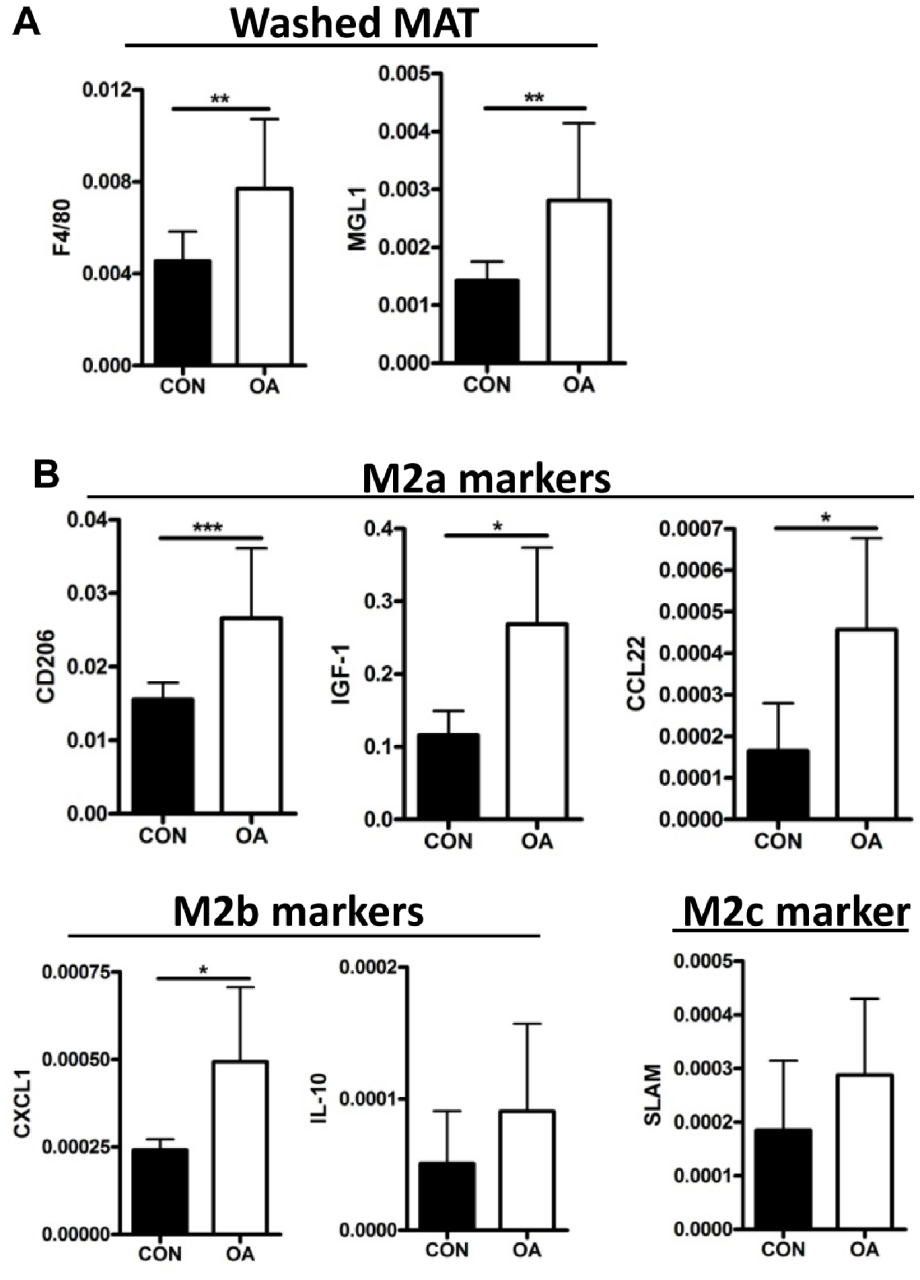
Dietary oleic acid increases M2 markers in the washed MAT. Gene expression analysis for (A) F4/80 and MGL1, (B) subsets of M2 macrophage markers (qPCR, relative to Gapdh), in washed MAT from mice given control gavage or oleic acid gavage twice daily for three days. (N = 10, 12).

To identify if OA-increases in M2 markers were intrinsic within the adipose tissue, mice were given control or OA gavage for three days. MAT was thoroughly washed to remove all adherent cells. Similar to results from unwashed tissue (Figure 3C), there were significant increases in macrophage markers F4/80 and MGL1 (Figure 7A). OA-gavaged mice also showed increases in M2a and M2b markers: CD206, IGF-1, CCL22 and CXCL1, but not IL-10 or M2c marker SLAM (Figure 7B).

## Discussion

In this report, we have provided data supporting the conclusion that dietary oleic acid increases M2 macrophages intrinsic to the MAT. We have seen that both oral gavage and direct culture with oleic acid increased M2 macrophage markers. A diet containing oleic acid induced significant changes in two distinct populations of F4/80^+^ macrophages within the abdominal cavity. One population, (F4/80 intermediate), is resident within the MAT, and significantly increases in expression of the M2 macrophage marker, CD206. Another population, within the peritoneal cavity, F4/80^HI^CD11b^HI^, apparently undergoes a significant increase in adhesiveness to the MAT that is ICAM-1 dependent, but not dependent upon oleic acid.

Using several different approaches, we have shown that oleic acid increases macrophage expression of M2 markers. In vitro culture and oral gavage with oleic acid increased Arg1, CD206 and MGL1 expression levels. In agreement with this data, ad lib intake of a high fat diet increased expression of CD206 on F4/80^INT^ macrophages resident within the MAT. There was no increase in total F4/80^+^CD11b^+^ cells/g AT (data not shown), indicating these cells are not migrating in, but instead are changing their expression pattern. These data are consistent with previous evidence that oleic acid induces a protective, anti-inflammatory response. In culture, oleic acid inhibits trans-conjugated linoleic acid-induced inflammatory gene expression in adipocytes [10] and inhibits TNFα-induced oxidative stress in cardiomyocytes [21]. Dermal or oral application of oleic acid to a wound area has been shown to accelerate healing [22], [23], and the ability of olive oil to reduce blood pressure appears to be due to the high oleic acid content [24]. Classically, M2 macrophages are considered anti-inflammatory and have roles in wound healing and tissue growth [2]. Considering that an increase in fat mass was identified at three days of HMF feeding, it is possible that one role for the enhanced M2 polarization in response to the diet is to promote vascular and adipocyte growth, allowing for proper lipid storage.

Palmitic acid is present at a higher amount in milk-fat, but oleic acid is higher within the MAT. This preferential storage for oleic acid may be the reason why the HMF elicits an anti-inflammatory response within the MAT, but not the EAT, despite the presence of palmitic acid within the diet. When purified palmitic acid was given by oral gavage, there was no change in M1 or M2 macrophage markers in the MAT. This was particularly surprising and contradicted ours, and previous data, which showed that in vitro culture with palmitate induced TNFα expression in macrophages [13], [25]. Palmitic acid is a well-known inflammatory mediator in culture and in long term feeding studies [3], [26], [27], but the short term effects of dietary palmitic acid are less characterized. Although palmitic acid has no identified effects on M1 or M2 genes in the MAT when ingested alone, its presence within the HMF diet may clarify the dampened anti-inflammatory response to HMF diet when comparing it to direct supplementation with oleic acid. In vitro, we found that palmitic acid completely inhibited the oleic acid-induced increase in M2 macrophage markers. The inhibitory effects of oleic acid upon palmitic acid-induced inflammation have been established [28]; apparently the mixture of the two fatty-acids produces an overall dampened response as compared to a single fatty acid alone. Our results in vivo and in vitro are not in complete agreement (partial inhibition versus complete inhibition of M2 markers), but this is likely a result of the more complex cellular and metabolic environment present in vivo.

One potential mechanism for oleic acid activity is through its activation of nuclear receptors from the peroxisome proliferator-activated receptor (PPAR) family, which controls nearly every component of fatty acid metabolism, including transport, synthesis, storage, mobilization and oxidation of fatty acids [11], [29], [30]. PPARγ is abundantly expressed in macrophages, where its activity promotes M2 polarization by increasing oxidative metabolism, as opposed to the switch to glycolysis that is seen in LPS-stimulated M1 macrophages, by increasing the expression of anti-inflammatory genes and by inhibiting the activity of NFκB and transcription of its target genes [12], [13], [31]. Macrophage-expression of PPARγ is required to maintain adipose tissue homeostasis and insulin sensitivity, as mice with a macrophage-specific deletion of PPARγ are insulin resistant, even when on a control diet, and have aggravated adiposity and adipose tissue inflammation when placed on a high fat diet containing high amounts of oleic acid [12]. Oleic acid-induced triglyceride uptake in hepatic cells was prevented in PPARγ-deficient cells [32]. Our data demonstrates the ability of oleic acid to alter macrophage polarization; based on the expression level and activities of PPARγ, it would be a candidate receptor to mediate oleic acid-induced macrophage polarization.

In addition to finding a short- term HMF diet-induced increases in the resident adipose tissue macrophages, we identified increased adherence by peritoneal macrophages to MAT. Our results indicate that oleic acid is not sufficient to induce peritoneal macrophage adherence, as evidenced by failure to wash away F4/80 increase following oleic acid gavage (Figure 7A). Adherent macrophages express high levels of the macrophage markers F4/80 and CD11b. It is not clear what type of macrophages the F4/80^HI^CD11b^HI^ macrophages are, as they do not express M1 (CD11c) or M2 (CD206) macrophage markers. They express a high level of CD11b, a ligand for ICAM-1 [33], suggesting that the interaction between CD11b and ICAM-1 may mediate macrophage adherence. Mesenteric adipose is unique in that it is covered in a layer of mesothelial cells which express ICAM-1 and vascular cell adhesion protein (VCAM)-1 for direct interaction with peritoneal cells [34], [35]. ICAM-1 deficient mice fail to upregulate F4/80 following HMF feeding, suggesting that mesothelial expression of ICAM-1 may be responsible for macrophage interaction with MAT. Previous studies have shown leukocyte interaction and migration across the mesothelium occurring following peritoneal infection [36]. This interaction and migration requires both adhesion molecules and chemokine gradient to recruit the cells to the proper location [37], [38]. Our results show that mice deficient in MCP-1 have a smaller increase in F4/80 gene expression following HMF feeding, as compared to wild-type gene- increase. The difference is nearly significant (P = 0.05), indicating the MCP-1 may play a role in diet-induced macrophage adherence. While the dynamic activities of tissue macrophages in response to high fat diets are clear [1], [2], [39], this is the first suggestion that peritoneal macrophages interact with adipose tissue as a result of high fat feeding.

These data and previous reports indicate that dietary fatty-acids can regulate adipose tissue macrophage polarization. Furthermore, short term mixed-fat feeding shows an anti-inflammatory response and this response is distinct from the systemic inflammation seen in long term feeding studies.

## References

[1] Lumeng CN, Bodzin JL, Saltiel AR (2007) Obesity induces a phenotypic switch in adipose tissue macrophage polarization. J Clin Invest 117: 175–184. 17200717 10.1172/JCI29881 PMC1716210

[2] Odegaard JI, Chawla A (2011) Alternative macrophage activation and metabolism. Annu Rev Pathol 6: 275–297. 21034223 10.1146/annurev-pathol-011110-130138 PMC3381938

[3] Davis JE, Gabler NK, Walker-Daniels J, Spurlock ME (2008) Tlr-4 deficiency selectively protects against obesity induced by diets high in saturated fat. Obesity (Silver Spring) 16: 1248–1255. 18421279 10.1038/oby.2008.210

[4] Shi H, Kokoeva MV, Inouye K, Tzameli I, Yin H, et al (2006) TLR4 links innate immunity and fatty acid-induced insulin resistance. J Clin Invest 116: 3015–3025. 17053832 10.1172/JCI28898 PMC1616196

[5] Burdge GC, Calder PC (2005) Plasma cytokine response during the postprandial period: a potential causal process in vascular disease? Br J Nutr 93: 3–9. 15705218 10.1079/bjn20041282

[6] Lanthier N, Molendi-Coste O, Horsmans Y, van Rooijen N, Cani PD, et al (2010) Kupffer cell activation is a causal factor for hepatic insulin resistance. Am J Physiol Gastrointest Liver Physiol 298: G107–G116. 19875703 10.1152/ajpgi.00391.2009

[7] Elgazar-Carmon V, Rudich A, Hadad N, Levy R (2008) Neutrophils transiently infiltrate intra-abdominal fat early in the course of high-fat feeding. J Lipid Res 49: 1894–1903. 18503031 10.1194/jlr.M800132-JLR200

[8] Ji Y, Sun S, Xia S, Yang L, Li X, et al (2012) Short term high fat diet challenge promotes alternative macrophage polarization in adipose tissue via natural killer T cells and interleukin-4. J Biol Chem 287: 24378–24386. 22645141 10.1074/jbc.M112.371807 PMC3397864

[9] Maedler K, Oberholzer J, Bucher P, Spinas GA, Donath MY (2003) Monounsaturated fatty acids prevent the deleterious effects of palmitate and high glucose on human pancreatic beta-cell turnover and function. Diabetes 52: 726–733. 12606514 10.2337/diabetes.52.3.726

[10] Reardon M, Gobern S, Martinez K, Shen W, Reid T, et al (2012) Oleic Acid Attenuates trans-10,cis-12 Conjugated Linoleic Acid-Mediated Inflammatory Gene Expression in Human Adipocytes. Lipids 47: 1043–1051. 22941440 10.1007/s11745-012-3711-0 PMC3479322

[11] Kliewer SA, Sundseth SS, Jones SA, Brown PJ, Wisely GB, et al (1997) Fatty acids and eicosanoids regulate gene expression through direct interactions with peroxisome proliferator-activated receptors alpha and gamma. Proc Natl Acad Sci U S A 94: 4318–4323. 9113987 10.1073/pnas.94.9.4318 PMC20720

[12] Odegaard JI, Ricardo-Gonzalez RR, Goforth MH, Morel CR, Subramanian V, et al (2007) Macrophage-specific PPARgamma controls alternative activation and improves insulin resistance. Nature 447: 1116–1120. 17515919 10.1038/nature05894 PMC2587297

[13] Bouhlel MA, Derudas B, Rigamonti E, Dievart R, Brozek J, Haulon S, et al (2007) PPARgamma activation primes human monocytes into alternative M2 macrophages with anti-inflammatory properties. Cell Metab 6: 137–143. 17681149 10.1016/j.cmet.2007.06.010

[14] Brake DK, Smith CW (2008) Flow cytometry on the stromal-vascular fraction of white adipose tissue. Methods Mol Biol 456: 221–229. 18516564 10.1007/978-1-59745-245-8_16

[15] Weischenfeldt J, Porse B (2008) Bone Marrow-Derived Macrophages (BMM): Isolation and Applications. CSH Protoc 2008: db. 10.1101/pdb.prot5080 21356739

[16] Benoit SC, Kemp CJ, Elias CF, Abplanalp W, Herman JP, et al (2009) Palmitic acid mediates hypothalamic insulin resistance by altering PKC-theta subcellular localization in rodents. J Clin Invest 119: 2577–2589. 19726875 10.1172/JCI36714 PMC2735917

[17] Prieur X, Mok CY, Velagapudi VR, Nunez V, Fuentes L, et al (2011) Differential lipid partitioning between adipocytes and tissue macrophages modulates macrophage lipotoxicity and M2/M1 polarization in obese mice. Diabetes 60: 797–809. 21266330 10.2337/db10-0705 PMC3046840

[18] Huang S, Rutkowsky JM, Snodgrass RG, Ono-Moore KD, Schneider DA, et al (2012) Saturated fatty acids activate TLR-mediated proinflammatory signaling pathways. J Lipid Res 53: 2002–2013. 22766885 10.1194/jlr.D029546 PMC3413240

[19] Turchyn LR, Baginski TJ, Renkiewicz RR, Lesch CA, Mobley JL (2007) Phenotypic and functional analysis of murine resident and induced peritoneal macrophages. Comp Med 57: 574–580. 18246870

[20] Brake DK, Smith EO, Mersmann H, Smith CW, Robker RL (2006) ICAM-1 expression in adipose tissue: effects of diet-induced obesity in mice. Am J Physiol Cell Physiol 291: C1232–C1239. 16807303 10.1152/ajpcell.00008.2006

[21] Al Shudiefat AA, Sharma AK, Bagchi AK, Dhingra S, Singal PK (2013) Oleic acid mitigates TNF-alpha-induced oxidative stress in rat cardiomyocytes. Mol Cell Biochem 372: 75–82. 22961439 10.1007/s11010-012-1447-z

[22] Cardoso CR, Favoreto S Jr, Oliveira LL, Vancim JO, Barban GB, et al (2011) Oleic acid modulation of the immune response in wound healing: a new approach for skin repair. Immunobiology 216: 409–415. 20655616 10.1016/j.imbio.2010.06.007

[23] Mensink RP, Zock PL, Kester AD, Katan MB (2003) Effects of dietary fatty acids and carbohydrates on the ratio of serum total to HDL cholesterol and on serum lipids and apolipoproteins: a meta-analysis of 60 controlled trials. Am J Clin Nutr 77: 1146–1155. 12716665 10.1093/ajcn/77.5.1146

[24] Teres S, Barcelo-Coblijn G, Benet M, Alvarez R, Bressani R, et al (2008) Oleic acid content is responsible for the reduction in blood pressure induced by olive oil. Proc Natl Acad Sci U S A 105: 13811–13816. 18772370 10.1073/pnas.0807500105 PMC2544536

[25] Haversen L, Danielsson KN, Fogelstrand L, Wiklund O (2009) Induction of proinflammatory cytokines by long-chain saturated fatty acids in human macrophages. Atherosclerosis 202: 382–393. 18599066 10.1016/j.atherosclerosis.2008.05.033

[26] Karaskov E, Scott C, Zhang L, Teodoro T, Ravazzola M, et al (2006) Chronic palmitate but not oleate exposure induces endoplasmic reticulum stress, which may contribute to INS-1 pancreatic beta-cell apoptosis. Endocrinology 147: 3398–3407. 16601139 10.1210/en.2005-1494

[27] Laine PS, Schwartz EA, Wang Y, Zhang WY, Karnik SK, et al (2007) Palmitic acid induces IP-10 expression in human macrophages via NF-kappaB activation. Biochem Biophys Res Commun 358: 150–155. 17467667 10.1016/j.bbrc.2007.04.092

[28] Listenberger LL, Han X, Lewis SE, Cases S, Farese RV Jr, et al (2003) Triglyceride accumulation protects against fatty acid-induced lipotoxicity. Proc Natl Acad Sci U S A 100: 3077–3082. 12629214 10.1073/pnas.0630588100 PMC152249

[29] Kersten S, Desvergne B, Wahli W (2000) Roles of PPARs in health and disease. Nature 405: 421–424. 10839530 10.1038/35013000

[30] Chawla A (2010) Control of macrophage activation and function by PPARs. Circ Res 106: 1559–1569. 20508200 10.1161/CIRCRESAHA.110.216523 PMC2897247

[31] Remels AH, Langen RC, Gosker HR, Russell AP, Spaapen F, et al (2009) PPARgamma inhibits NF-kappaB-dependent transcriptional activation in skeletal muscle. Am J Physiol Endocrinol Metab 297: E174–E183. 19417127 10.1152/ajpendo.90632.2008

[32] Moran-Salvador E, Lopez-Parra M, Garcia-Alonso V, Titos E, Martinez-Clemente M, et al (2011) Role for PPARgamma in obesity-induced hepatic steatosis as determined by hepatocyte- and macrophage-specific conditional knockouts. FASEB J 25: 2538–2550. 21507897 10.1096/fj.10-173716

[33] Diamond MS, Staunton DE, de Fougerolles AR, Stacker SA, Garcia-Aguilar J, et al (1990) ICAM-1 (CD54): a counter-receptor for Mac-1 (CD11b/CD18). J Cell Biol 111: 3129–3139. 1980124 10.1083/jcb.111.6.3129 PMC2116396

[34] Muller J, Yoshida T (1995) Interaction of murine peritoneal leukocytes and mesothelial cells: in vitro model system to survey cellular events on serosal membranes during inflammation. Clin Immunol Immunopathol 75: 231–238. 7768040 10.1006/clin.1995.1076

[35] Jonjic N, Peri G, Bernasconi S, Sciacca FL, Colotta F, et al (1992) Expression of adhesion molecules and chemotactic cytokines in cultured human mesothelial cells. J Exp Med 176: 1165–1174. 1383376 10.1084/jem.176.4.1165 PMC2119405

[36] Topley N, Mackenzie RK, Williams JD (1996) Macrophages and mesothelial cells in bacterial peritonitis. Immunobiology 195: 563–573. 8933157 10.1016/S0171-2985(96)80022-2

[37] van Rossen ME, Hofland LJ, van den Tol MP, van Koetsveld PM, Jeekel J, et al (2001) Effect of inflammatory cytokines and growth factors on tumour cell adhesion to the peritoneum. J Pathol 193: 530–537. 11276014 10.1002/1096-9896(2000)9999:9999<::AID-PATH805>3.0.CO;2-O

[38] Li FK, Davenport A, Robson RL, Loetscher P, Rothlein R, et al (1998) Leukocyte migration across human peritoneal mesothelial cells is dependent on directed chemokine secretion and ICAM-1 expression. Kidney Int 54: 2170–2183. 9853284 10.1046/j.1523-1755.1998.00174.x

[39] Morris DL, Singer K, Lumeng CN (2011) Adipose tissue macrophages: phenotypic plasticity and diversity in lean and obese states. Curr Opin Clin Nutr Metab Care 14: 341–346. 21587064 10.1097/MCO.0b013e328347970b PMC4690541

